# Entropy generation in bioconvection nanofluid flow between two stretchable rotating disks

**DOI:** 10.1038/s41598-020-61172-2

**Published:** 2020-03-10

**Authors:** Noor Saeed Khan, Qayyum Shah, Amiya Bhaumik, Poom Kumam, Phatiphat Thounthong, Irajsadegh Amiri

**Affiliations:** 1 Department of Mathematics, Abdul Wali Khan University, Mardan, 23200 Khyber Pakhtunkhwa Pakistan; 2 Department of Basic Sciences and Islamiyat, University of Engineering & Technology, Peshawar, 25000 Khyber Pakhtunkhwa Pakistan; 3 Faculty of Engineering, Lincoln University College (LUC), Lincoln, 1440 Malaysia; 4 KMUTTFixed Point Research Laboratory, Room SCL 802 Fixed Point Laboratory, Science Laboratory Building, Department of Mathematics, Faculty of Science, King Mongkut’s University of Technology Thonburi (KMUTT), Bangkok, 10140 Thailand; 5 KMUTT-Fixed Point Theory and Applications Research Group, Theoretical and Computational Science Center (TaCS), Science Laboratory Building, Faculty of Science, King Mongkut’s University of Technology Thonburi (KMUTT), Bangkok, 10140 Thailand; 6 Department of Medical Research, China Medical University Hospital, China Medical University, Taichung, 40402 Taiwan; 7 Renewable Energy Research Center, Department of Teacher Training in Electrical Engineering, Faculty of Technical Education, King Mongkut’s University of Technology North Bangkok, 1518, Wongsawang, Bangsue, Bangkok, 10800 Thailand; 8 Computational Optics Research Group, Advanced Institute of Materials Science, Ton Duc Thang University, Ho Chi Minh City, 700000 Vietnam; 9 Faculty of Applied Sciences, Ton Duc Thang University, Ho Chi Minh City, 700000 Vietnam

**Keywords:** Mathematics and computing, Physics

## Abstract

Buongiorno’s nanofluid model is followed to study the bioconvection in two stretchable rotating disks with entropy generation. Similarity transformations are used to handle the problem equations for non-dimensionality. For the simulation of the modeled equations, Homotopy Analysis Method is applied. The biothermal system is explored for all the embedded parameters whose effects are shown through different graphs. There exists interesting results due to the effects of different parameters on different profiles. Radial velocity decreases with increasing stretching and magnetic field parameters. Temperature increases with Brownian motion and thermophoresis parameters. Nanoparticles concentration decreases on increasing Lewis number and thermophoresis parameter while motile gyrotactic microorganisms profile increases with increasing Lewis and Peclet numbers. Convergence of the solution is found and good agreement is obtained when the results are compared with published work.

## Introduction

Natural convection has an outstanding applications in daily life. These applications are exist in petrochemical processes, cooling of electronic components, geothermal engineering, crystal growth processes, in the annular gap between the rotor and stator, thermal insulation system, food industry, growth of single silicon crystals, packed bed chemical reactors, grain storage installations, rotating systems, porous heat exchangers, fuel cells, solar ponds etc. Researchers paid extensive attention to work on convection. Venkatachalappa *et al*.^1^ performed a study to analyze the role of rotation on the axisymmetric gravity driven complex flow in a cylindrical annulus whose side walls rotate about their axis with different angular velocities. They obtained the results for Grashof number, rotational speeds, Prandtl number, aspect ratio and compared the results with the existing data. Khan *et al*.^2^ treated the movement in heating prevailing system of a differential type dispersion on an expanding medium using series solution. Sankar *et al*.^3^ tested numerically the hydromagnetic field influence in axial or radial forms for natural convection of a low Prandtl number electrically conducting fluid in a vertical cylindrical annulus. Their outcomes showed that in shallow cavities the flow and heat transfer were suppressed sufficiently through an axial magnetic field and in tall cavities the radial magnetic field had an excellent output. Khan *et al*.^4^ tested the thermal disorder, heat and mass transfer tiny dispersion movement with gyrotactic microorganisms in porous medium using heating wall information. Using the Brinkman-extended Darcy equation, Sankar *et al*.^5^ investigated the natural convection flows in a vertical annulus filled with a fluid-saturated porous medium in which the inner wall was subjected to discrete heating, outer wall was subjected to isothermally at lower temperature and the adiabatic parts were the bottom and top walls including the unheated regions of the inner wall. They applied the finite difference method and observed that enhanced heat transfer exist by placing the heater in lower half of the inner wall. Zuhra *et al*.^6^ presented the work on gravity driven simultaneous flow of Casson and Williamson nanofluids and heat transfer with homogeneous-heterogeneous chemical reactions. Sankar *et al*.^7^ attempted numerically the natural convection heat transfer in a cylindrical annular cavity with discrete heat sources on the inner wall whose purpose is to cool the chips in an effective way to prevent overheating and hot spots. Khan *et al*.^8^ followed the Buongiorno’s nanodisperion concept to model mathematically the biconvection in nanodispersion transmission in stretchable object persisting porous space, Arrhenius activation energy and binary chemical reaction. Sankar *et al*.^9^ documented a report on the double diffusive convection in a vertical annulus filled with a fluid-saturated porous medium accompanying the effects of discrete source of heat and solute on the fluid flow, heat and mass transfer rates in which the location of stronger flow circulation is independent of the higher heat and mass transfer rates in the porous region. Zuhra *et al*.^10^ used the OHAM solution to develop the special form of initial value problems to complex KdV equation in which three different types of semi analytic complextion solutions from complex KdV equation have been achieved. Sankar *et al*.^11^ carried out the work on Brinkman extended Darcy equation for the natural convection heat transfer due to two discrete heat sources showing that the bottom heater is found to dissipate higher heat transfer compared to top heater. The convection and saturation transferring studies may be consulted in the references^12–24^.

Nanofluids contain the suspended nanoparticles whose diameters are less than 100 nm, used for the enhanced thermal conductivity. Nanofluids have applications in solar water heating, improving transportation, heat transfer efficiency of refrigerator and chillers, and optimal absorption of solar energy. Nanofluids are used for the cooling of machine equipments, nuclear reactor, transformer oil, and microelectronics. These are also used in drugs delivery and radiation in patients. The first innovative work in this regard is due to Choi^25^ whose work pawed the way for researchers to investigate nanofluids. Irfan *et al*.^26^ presented the impact of chemical reaction and activation energy on dual nature of unsteady flow of Carreau magnetite nanofluid owing to shrinking/stretching sheet in the presence of convective conditions, thermal radiation, viscous dissipation, Joule heating and heat source/sink. Hashim *et al*.^27^ provided a novel study to develop and understand a mathematical model for a non-Newtonian Williamson fluid taking into account the nanoparticles which described the thermal characteristics of nanofluid through Rosseland approximation to illustrate the nonlinear radiation effects. Moradi *et al*.^28^ projected an experimental investigation on heat transfer characteristics of multi-walled carbon nanotube aqueous nanofluids inside a countercurrent double-pipe heat exchanger using porous media. They used the aluminum porous media due to the construction of the medium, with porous plate media at the center of the inner tube and with three porous plates on the walls of the inner tube for investigating the effects of parameters like flow rate, mass fraction of nanofluids, and inlet temperature of nanofluids. Sadiq *et al*.^29^ inquired the properties of MHD oscillatory oblique stagnation flow of micropolar fluid immersed with Cu and Al_2_O_3_ assuming magnetic field parallel towards the isolating streamline to model both of weak and strong concentration. In that paper, it is proved that magnetic effect is prominent on Cu compared to Al_2_O_3_. Benos *et al*.^30^ studied the laminar two-dimensional MHD natural convection in a shallow cavity using a carbon nanotube water nanofluid which is internally heated by volumetrically heat sources, exploring an interfacial nanolayer adjacent to solid particles and a nutshell where increasing the concentration of the carbon nanotube generated the decrement in fluid flow. Ramzan *et al*.^31^ solved the problem of three dimensional MHD couple stress nanofluid flow with Joule heating and viscous dissipation past an exponential stretching surface taking into account Brownian motion and thermophoresis effects with convective heat condition which distinctly introduced a realistic boundary constraint for nanofluid flow model.

Rotating flows have applications in computer disk drives, mass spectromentries, jet motors, electric power generating and turbine systems, and food processing. Rout *et al*.^32^ analyzed the axisymmetric flows of copper and silver water nanofluids between two rotating disks in the presence of Hartmann number, porous medium, and drag coefficient with thermal radiation. They used the Adomian Decomposition Method (ADM) to solve the coupled ordinary differential equations and proved that an enhancement in solid volume fraction decreased the velocity. Ahmad *et al*.^33^ investigated the Maxwell nanofluid flow between two coaxially parallel stretchable rotating disks in the presence of axial magnetic field and variable thermal conductivity. They used the Buongiorno nanofluid model and showed the behaviors of upper and lower disks in the same and opposite directions. Li *et al*.^34^ reported a three-dimensional unsteady mixed nano-bioconvection flow between two contracting or expanding rotating disks using the passively controlled nanofluid model in which the Brownian diffusion and thermophoresis were considered as the two dominant factors for nanoparticles/base-fluid slip mechanisms. Hayat *et al*.^35^ explored the flow between two stretchable rotating disks in porous medium with Cattaneo-Christov heat flux theory finding that motion in *y*-direction decreased with in increase in rotational parameter. Ahmed *et al*.^36^ used the Von Karman similarity transformations for the Buongiorno’s nanofluid model and incorporated the revised condition for nanoparticle volume fraction implementing finite difference technique known as Keller box method for the solution of the problem.

Thermodynamics second law is equally useful like the first law. The second law analysis is effectively used in heat transfer mechanisms. It is used in minimizing the irreversibility of thermal systems. Various authors have discussed entropy generation. For example, Abbas *et al*.^37^ discussed the entropy generation in peristaltic flow of nanofluids in a non-uniform two dimensional channel with compliant walls whose mathematical modeling was obtained under the approximation of long wavelength and zero Reynolds number. Khan *et al*.^38^ followed the Tiwari-Das model of nanofluid for the flow of aluminum and copper nanoparticles between two rotating disks to discuss the entropy generation, statistical declaration and probable error in the presence of Joule heating and thermal radiation. Al-Rashed *et al*.^39^ investigated the nanoparticle shapes linked to entropy generation of boehmite alumina nanoparticles of different shapes (cylindrical, brick, blade, platelet and spherical) dispersed in a mixture of water/ethylene glycol flowing through a horizontal double-pipe minichannel heat exchanger to prove that platelet shape nanoparticles had the high entropy generation compared to spherical shape. Shukla *et al*.^40^ presented the theoretical study of multiple slip flow with entropy generation in mixed convection MHD flow of an electrically conducting nanofluid on a vertical cylinder with viscous dissipation, no-flux nanoparticle concentration resulting that entropy increased with second order velocity slip, magnetic field and curvature parameter. Rashidi *et al*.^41^ studied entropy generation on MHD blood flow caused by peristaltic waves employing perturbation method for the solution of the problem stating that the study is applied in fluids pumping for pulsating and non-pulsating continuous motion in different channels structure as well as controlling the flow. Madiha *et al*.^42^ analyzed five nanoparticles namely silver, copper, copper oxide, titanium oxide, and aluminum oxide with water as base fluid for the entropy generation on stretching cylinder with nonlinear radiation, non-uniform heat source/sink, convective conditions and Darcy-Forchheimer relation showing that entropy generation depended on Brinkman number, temperature difference parameter and Forchheimer number. Rashidi *et al*.^43^ compared the single and two phase modeling approaches for force convective turbulent flow for TiO_2_ nanoparticles of spherical shape with water as base fluid in a horizontal tube with constant wall heat flux boundary condition. Their output showed that the entropy generation for thermal and turbulent dissipation were very close to single-phase and mixture models. Selimefendigil and Oztop^44^ worked on a vented cavity with inlet and outlet ports investigating mixed convection and entropy generation using an inclined magnetic field where the numerical simulation was performed for various values of Reynolds number, Hartmann number and solid volume fractions of CuO nanoparticles. They used the Galerkin weighted finite element method to evaluate the solution achieving different results for different parts of the cavity, Hartmann number and entropy generation. Rashidi *et al*.^45^ considered the analysis of the second law of thermodynamics applied to an electrically conducting incompressible nanofluid flowing past a porous rotating disk in the presence of an externally applied uniform vertical magnetic field. They stated that the simulation of the problem has applications in novel nuclear space propulsion engines, heat transfer enhancement in renewable energy systems and industrial thermal management.

At present convection through motile microorganisms is growing high response on account of their uses in microfluidic devices, like biogalvanic devices and biosciences dispersions and in the investigation of few species of thermophiles existing in springs having high temperature, in microbial oil recovery, and in formulation of oil and gas carrying sedimentary basins. Khan *et al*.^46^ reported a study to investigate the bioconvection due to gyrotactic microorganisms and nanoparticles which showed that conduction increases with increasing the buoyancy parameter in the presence of convective condition while at the same time nanoparticle concentration increased with the enhancement of Brownian motion parameter. De^47^ obtained the dual solutions for water based nanofluid and gyrotactic microorganisms with thermal radiation on nonlinear shrinking/stretching sheet. Using fifth order Runge-Kutta-Fehlberg method along with shooting method for solution, his findings revealed that motile microorganisms function decreased for the enhancement of bioconvection Lewis number. Palwasha *et al*.^48^ presented a study that considered the gravity driven nanofluid flow containing nanoparticles and gyrotactic microorganisms. They solved the problem through Homotopy Analysis Method and explored that the simultaneous motion of Casson and Williamson nanofluids decreased with high magnetic field parameter. On the slip side, Khan *et al*.^49^ obtained the results of fluid flow, heat transfer containing nanoparticles and gyrotactic microorganisms in the presence of non-Newtonian nanofluids. They showed that nanofluids flow, heat transfer, nanoparticles and gyrotactic microorganisms concentrations had realistic results for passively controlled nanofluid model boundary conditions compared to the actively controlled nanofluid model boundary conditions. Zuhra *et al*.^50^ analyzed gyrotactic microorganisms and nanoparticles along with second grade nanofluid flow and heat transfer in which temperature increased with thermophoresis parameter.

Motivated from the above important investigations, the present study analyzes the entropy generation, flow, heat transfer, nanoparticles and gyrotactic microorganisms concentration via Homotopy Analysis Method^51^ solution. Graphs are sketched to show the influences of all parameters on different profiles.

## Methods

### Problem formulation

**Figure 1:**
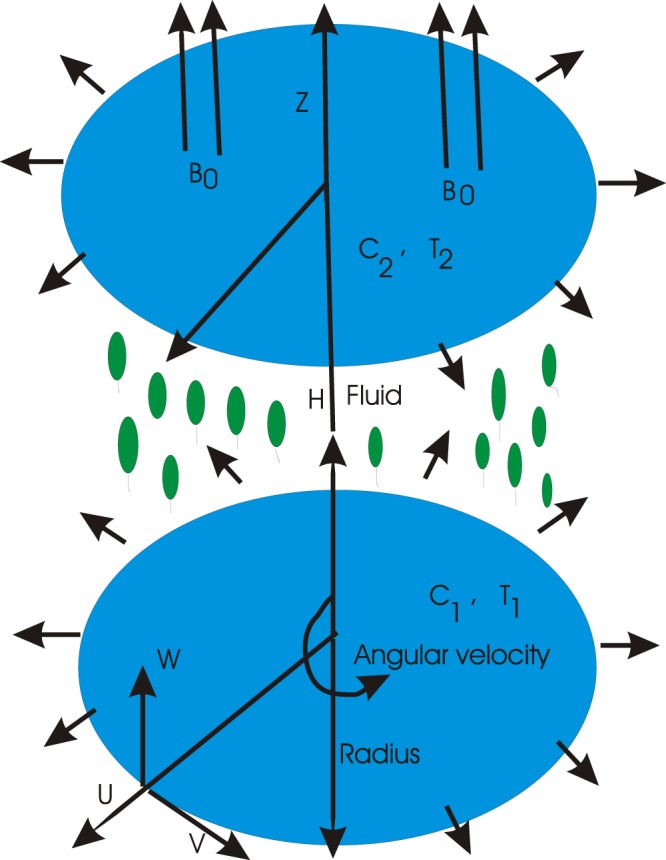
Geometry of the problem.

The axisymmetric motion of magnetohydrodynamic three dimensional, time independent and an incompressible nanofluid between two parallel infinite disks is considered. The lower disk is supposed to lie at *z* = 0. The distance between upper and lower disks is *H*. It is important to note that the lower and upper disks have the angular velocities Ω_1_ and Ω_2_ respectively in the rotation of axial direction. The stretching, temperature, concentration values on these disks are respectively *a*_1_, *T*_1_, *C*_1_ and *a*_2_, *T*_2_, *C*_2_. An intensified magnetic field of strength *B*_0_ is applied in the *z*-direction (see Fig. 1).

The water is taken as base fluid with nanoparticles. The nanoparticles volume fraction on both of the disks are satisfied with the actively controlled model *i. e*. there exist the nanoparticle flux at the walls. The distributions of motile gyrotactic microorganisms on the lower and upper disks are *N*_1_ and *N*_2_ respectively. The dilution of nanofluid is assumed to prevent the bioconvection instability on behalf of dispersion viscosity. Assumption is also taken that the nanoparticles suspended in the base fluid are stable which have no effect on the swimming direction and velocity of the microorganisms.

Keeping in mind the aforementioned conditions, the following five field equations carrying the conservation of total mass, momentum, thermal energy, nanoparticle volume fraction, and microorganisms are given as in^34^

1 $$\nabla \cdot {\bf{v}}=0,$$

2 $${\rho }_{nf}({\bf{v}}\cdot \nabla )\nabla \cdot {\bf{v}}=-\nabla p+{\mu }_{nf}{\nabla }^{2}{\bf{v}}+{\bf{J}}\times {\bf{B}},$$

3 $${(\rho {c}_{P})}_{nf}({\bf{v}}\cdot \nabla )T=\alpha {\nabla }^{2}T+\tau \left[{D}_{B}\nabla T\cdot \nabla C+\left(\frac{{D}_{T}}{{T}_{2}}\right)\nabla T\cdot \nabla T\right],$$

4 $$({\bf{v}}\cdot \nabla )C={D}_{B}{\nabla }^{2}C+\left(\frac{{D}_{T}}{{T}_{2}}\right){\nabla }^{2}T,$$

5 $$\nabla \cdot {\bf{j}}=0,$$

where **j** is defined as

6 $${\bf{j}}=N{\bf{v}}+N{\tilde{v}}-{D}_{n}\nabla N,$$

also

7 $${\tilde{v}}=\left(\frac{b{W}_{c}}{\Delta C}\right)\nabla C.$$

Expanding Eqs. (1–5) in cylindrical coordinate system (*r*, *ϑ*, *z*), the driving formulations are as in^32–36,38^

8 $$\frac{\partial u}{\partial r}+\frac{u}{r}+\frac{\partial w}{\partial z}=0,$$

9 $${\rho }_{nf}\left[u\frac{\partial u}{\partial r}+w\frac{\partial u}{\partial z}-\frac{{v}^{2}}{r}\right]=-\frac{\partial P}{\partial r}+{\mu }_{nf}\left[\frac{1}{r}\frac{\partial u}{\partial r}-\frac{u}{{r}^{2}}+\frac{{\partial }^{2}u}{\partial {r}^{2}}+\frac{{\partial }^{2}u}{\partial {z}^{2}}\right]-{\sigma }_{nf}{B}_{0}^{2}u,$$

10 $${\rho }_{nf}\left[u\frac{\partial v}{\partial r}+w\frac{\partial v}{\partial z}+\frac{uv}{r}\right]={\mu }_{nf}\left[\frac{1}{r}\frac{\partial v}{\partial r}-\frac{v}{{r}^{2}}+\frac{{\partial }^{2}v}{\partial {r}^{2}}+\frac{{\partial }^{2}v}{\partial {z}^{2}}\right]-{\sigma }_{nf}{B}_{0}^{2}v,$$

11 $${\rho }_{nf}\left[u\frac{\partial w}{\partial r}+w\frac{\partial w}{\partial z}\right]=-\frac{\partial P}{\partial z}+{\mu }_{nf}\left[\frac{1}{r}\frac{\partial w}{\partial r}+\frac{{\partial }^{2}w}{\partial {r}^{2}}+\frac{{\partial }^{2}w}{\partial {z}^{2}}\right],$$

12 $$\begin{array}{rcl}{(\rho {c}_{P})}_{nf}\left[u\frac{\partial T}{\partial r}+w\frac{\partial T}{\partial z}\right] & = & \alpha \left[\frac{1}{r}\frac{\partial T}{\partial r}+\frac{{\partial }^{2}T}{\partial {r}^{2}}+\frac{{\partial }^{2}T}{\partial {z}^{2}}\right]+\,\tau \left[{D}_{B}\left(\frac{\partial T}{\partial r}\frac{\partial C}{\partial r}+\frac{\partial T}{\partial z}\frac{\partial C}{\partial z}\right)+\frac{{D}_{T}}{{T}_{2}}\left\{{\left(\frac{\partial T}{\partial r}\right)}^{2}+{\left(\frac{\partial T}{\partial z}\right)}^{2}\right\}\right]\\  &  & \hspace{4.99878pt}+\,{\sigma }_{nf}{B}_{0}^{2}({u}^{2}+{v}^{2}),\end{array}$$

13 $$u\frac{\partial C}{\partial r}+w\frac{\partial C}{\partial z}={D}_{B}\left[\frac{1}{r}\frac{\partial C}{\partial r}+\frac{{\partial }^{2}C}{\partial {r}^{2}}+\frac{{\partial }^{2}C}{\partial {z}^{2}}\right]+\frac{{D}_{T}}{{T}_{2}}\left[\frac{1}{r}\frac{\partial T}{\partial r}+\frac{{\partial }^{2}T}{\partial {r}^{2}}+\frac{{\partial }^{2}T}{\partial {z}^{2}}\right],$$

14 $$w\frac{\partial N}{\partial z}+\widetilde{w}\frac{\partial N}{\partial z}+N\frac{\partial \widetilde{w}}{\partial z}={D}_{n}\frac{{\partial }^{2}N}{\partial {z}^{2}},$$

having boundary conditions

15 $$u=r{a}_{1},\ v=r{\Omega }_{1},\ w=0,\ T={T}_{1},\ C={C}_{1},\ N={N}_{1},\ at\ z=0,$$

16 $$u=r{a}_{2},\ v=r{\Omega }_{2},\ w=0,\ T={T}_{2},\ C={C}_{2},\ N={N}_{2}\ at\ z=H.$$

Following transformations are used

17 $$\begin{array}{l}u=r{\Omega }_{1}\,{f}^{{\prime} }(\zeta ),\ v=r{\Omega }_{1}g(\zeta ),\ w=-2H{\Omega }_{1}\,f(\zeta ),\ \theta (\zeta )=\frac{T-{T}_{2}}{{T}_{1}-{T}_{2}},\ \phi (\zeta )=\frac{C-{C}_{2}}{{C}_{1}-{C}_{2}},\\ h(\zeta )=\frac{N-{N}_{2}}{{N}_{1}-{N}_{2}},\ P={\rho }_{nf}{\Omega }_{1}{\nu }_{nf}\left[P(\zeta )+\frac{{r}^{2}\epsilon }{2{H}^{2}}\right],\ \zeta =\frac{z}{H}.\end{array}$$

Illustration of different mathematical letters and notations used in Eqs. (1–17) are given in Table 1. Substituting the values from Eq. (17) in Eqs. (9–16), generate the following eight Eqs. (18–25)

18 $$f{\rm{{\prime} }}{\rm{{\prime} }}{\rm{{\prime} }}+Re[2ff{\rm{{\prime} }}{\rm{{\prime} }}-f{{\rm{{\prime} }}}^{2}+{g}^{2}-Mf{\rm{{\prime} }}]-\epsilon =0,$$

19 $$g{\prime\prime} +Re[2fg{\prime} -Mg{\prime} ]=0,$$

20 $$P{\prime} =-\,4Reff{\prime} -f{\prime\prime} ,$$

21 $$\theta {\prime\prime} +PrRe[2f{\theta }^{{\prime} }+MEc({(f{\prime} )}^{2}+{g}^{2})]+Nb\theta {\prime} \phi {\prime} +Nt{(\theta {\prime} )}^{2}=0,$$

22 $$\phi {\prime\prime} +Re\left[2Lef\phi {\prime} +\frac{Nt}{Nb}\theta {\prime} \right]=0,$$

23 $$h{\prime\prime} +Re[2Scfh{\prime} +Pe(h{\prime} \phi {\prime} -h\phi {}^{{\prime\prime} })]=0,$$

24 $$f=0,\ f{\prime} ={k}_{1},\ g=1,\ \theta =1,\ \phi =1,\ h=1,\ P=0\ at\ \zeta =0,$$

25 $$f=0,\ f{\prime} ={k}_{2},\ g=\Omega ,\ \theta =0,\ \phi =0,\ h=0\ at\ \zeta =1.$$

The various parameters in Eqs. (18–25) are given in Table 2.

**Table 1:** Illustration of different mathematical letters and notations used in Eqs. (1–17).

Symbols/Notations	Illustration
**v** = (*u*, *v*, *w*)	Velocity of the nanofluid
**J**	Current density
**B**	Magnetic field
*C*	Nanoparticles concentration
*P*	Pressure
*D*_*B*_	Brownian diffusion coefficient
*D*_*T*_	Thermophoretic diffusion coefficient
**j**	Microorganisms flux
*N*	Density motile of microorganisms
*D*_*n*_	Microorganisms diffusion
*b*	Chemotaxic constant
*W*_*c*_	Maximum cell swimming speed
(*r*, *ϑ*, *z*)	Cylindrical coordinates
*H*	Distance between disks (m)
*u* (*r*, *ϑ*, *z*)	Velocity component
*v* (*r*, *ϑ*, *z*)	Velocity component
*w* (*r*, *ϑ*, *z*)	Velocity component
*B* = (0, 0, *B*_0_)	Magnetic induction
*T*	Temperature
*D*	Diffusion of species
*f*	Dimensionless velocity in radial direction
*g*	Dimensionless velocity in tangential direction
Greek symbols/Notations	Illustration
*ρ*_*nf*_	Density of nanofluid
*μ*_*nf*_	Dynamic viscosity of nanofluid
*σ*_*nf*_	Electrical conductivity of nanofluid
${(\rho {c}_{p})}_{nf}$	Heat capacity of nanofluid
${\tilde{v}}$	Average swimming velocity vector of the oxytactic microorganisms
*α*	Thermal diffusivity of nanofluid
*θ* (*ζ*)	Dimensionless temperature
*ϕ* (*ζ*)	Dimensionless concentration
*ν*_*nf*_	Kinematic viscosity of nanofluid
$\epsilon$	Pressure parameter
Subscripts	Illustration
nf	Nanofluid

**Table 2:** Various parameters in Eqs. (18–25).

Parameter names	Symbols/Notations	Defined values
Reynolds number	*Re*	$\frac{{\Omega }_{1}{H}^{2}}{{\nu }_{nf}}$
Magnetic field parameter	*M*	$\frac{{\sigma }_{nf}{B}_{0}^{2}}{{\rho }_{nf}{\Omega }_{1}}$
Prandtl number	*Pr*	$\frac{{({\rho }_{{c}_{P}})}_{nf}{\nu }_{nf}}{\alpha }$
Eckert number	*Ec*	$\frac{{r}^{2}{\Omega }_{1}^{2}}{{c}_{P}({T}_{1}-{T}_{2})}$
Schmidt number	*Sc*	$\frac{{\nu }_{nf}}{{D}_{n}}$
Lewis number	*Le*	$\frac{{\nu }_{nf}}{{D}_{B}}$
Bioconvection Peclet number	*Pe*	$\frac{b{W}_{c}}{{D}_{n}}$
Stretching parameter due to lower disk	*k*_1_	$\frac{{a}_{1}}{{\Omega }_{1}}$
Stretching parameter due to upper disk	*k*_2_	$\frac{{a}_{2}}{{\Omega }_{2}}$
Brownian motion parameter	*Nb*	$\frac{{D}_{B}({C}_{2}-{C}_{1})}{{\nu }_{nf}}$
Thermophoresis parameter	*Nt*	$\frac{\tau {D}_{T}({T}_{2}-{T}_{1})}{{\nu }_{nf}{T}_{1}}$
Rotation parameter	Ω	$\frac{{\Omega }_{2}}{{\Omega }_{1}}$
Superscripts	Illustration	
${}^{{\prime} }$	Differentiation with respect to *ζ*	

Differentiating Eq. (18) w. r. t. *ζ*

26 $$f{\prime\prime} {\prime\prime} +Re[2ff{\prime\prime} {\prime} +2gg{\prime} -Mf{\prime\prime} ]=0.$$

Accounting Eq. (18) and Eqs. (24,25), the pressure term $\epsilon$ becomes

27 $$\epsilon =f{\prime\prime} {\prime} (0)-Re[{(f{\prime} (0))}^{2}-{(g(0))}^{2}+Mf{\prime} (0)].$$

Solving Eq. (20) for *P* using integration for the range zero to *ζ*

28 $$P=-\,2[Re{(f)}^{2}+(f{\prime} -f{\prime} (0))].$$

### Important physical quantities

Proceeding for the local skin friction, Nusselt number, Sherwood number and motile microorganisms flux. On the lower and upper disks, the skin friction coefficients are respectively ${C}_{{f}_{1}}$ and ${C}_{{f}_{2}}$, defined as

29 $${C}_{{f}_{1}}=\frac{\tau {| }_{z=0}}{{\rho }_{nf}{(r{\Omega }_{1})}^{2}},\ {C}_{{f}_{2}}=\frac{\tau {| }_{z=H}}{{\rho }_{nf}{(r{\Omega }_{1})}^{2}},$$

where

30 $$\tau =\sqrt{{({\tau }_{zr})}^{2}+{({\tau }_{z\theta })}^{2}},$$

*τ* is the overall shear stress of shear stresses *τ*_*zr*_ and *τ*_*zθ*_ along radial and tangential directions respectively such that at lower disk

31 $${\tau }_{zr}={\mu }_{nf}\frac{\partial u}{\partial z}{| }_{z=0}=\frac{{\mu }_{nf}r{\Omega }_{1}f{\prime\prime} (0)}{H}\ and\ {\tau }_{z\theta }={\mu }_{nf}\frac{\partial v}{\partial z}{| }_{z=0}=\frac{{\mu }_{nf}r{\Omega }_{1}g{\prime} (0)}{H}.$$

Hence putting values in Eq. (29) from Eqs. (30) and (31), the lower and upper disks have the skin friction coefficients as

32 $${C}_{{f}_{1}}=\frac{1}{R{e}_{r}}{[{(f{\prime\prime} (0))}^{2}+{(g{\prime} (0))}^{2}]}^{\frac{1}{2}},$$

33 $${C}_{{f}_{2}}=\frac{1}{R{e}_{r}}{[{(f{\prime\prime} (1))}^{2}+{(g{\prime} (1))}^{2}]}^{\frac{1}{2}},$$

where $R{e}_{r}=r\frac{{\Omega }_{1}H}{{\nu }_{nf}}$ represents the local Reynolds number.

The local Nusselt numbers on the lower and upper disks are

34 $$N{u}_{{r}_{1}}=\frac{H{q}_{w}}{\alpha ({T}_{1}-{T}_{2})}{| }_{z=0},\ N{u}_{{r}_{2}}=\frac{H{q}_{w}}{\alpha ({T}_{1}-{T}_{2})}{| }_{z=H},$$

where *q*_*w*_ is wall heat flux and at lower disk it is

35 $${q}_{w}=-\,\alpha \frac{\partial T}{\partial z}{| }_{z=0}=-\alpha \frac{{T}_{1}-{T}_{2}}{H}\theta {\prime} (0).$$

Hence from Eq. (34), local Nusselt numbers on the lower and upper disks are

36 $$N{u}_{{r}_{1}}=-\theta {\prime} (0),\ N{u}_{{r}_{2}}=-\theta {\prime} (1).$$

The Sherwood numbers on the lower and upper disks are

37 $$S{h}_{{r}_{1}}=\frac{H{q}_{m}}{{D}_{B}({C}_{1}-{C}_{2})}{| }_{z=0},\ S{h}_{{r}_{2}}=\frac{H{q}_{m}}{{D}_{B}({C}_{1}-{C}_{2})}{| }_{z=H},$$

where *q*_*m*_ is wall mass flux and at lower disk it is

38 $${q}_{m}=-{D}_{B}\frac{\partial C}{\partial z}{| }_{z=0}=-{D}_{B}\frac{{C}_{1}-{C}_{2}}{H}\phi {\prime} (0).$$

Hence from Eq. (37), local Sherwood numbers on the lower and upper disks are

39 $$S{h}_{{r}_{1}}=-\phi {\prime} (0),\ S{h}_{{r}_{2}}=-\phi {\prime} (1).$$

The local motile microorganisms fluxes on the lower and upper disks are

40 $$S{n}_{{r}_{1}}=\frac{H{q}_{n}}{{D}_{n}({N}_{1}-{N}_{2})}{| }_{z=0},\ S{n}_{{r}_{2}}=\frac{H{q}_{n}}{{D}_{n}({N}_{1}-{N}_{2})}{| }_{z=H},$$

where *q*_*n*_ is wall motile microorganisms flux and at lower disk it is

41 $${q}_{n}=-\,{D}_{n}\frac{\partial N}{\partial z}{| }_{z=0}=-\,{D}_{n}\frac{{N}_{1}-{N}_{2}}{H}h{\prime} (0).$$

So from Eq. (40), local motile microorganisms fluxes on the lower and upper disks are

42 $$S{n}_{{r}_{1}}=-\,h{\prime} (0),\ S{n}_{{r}_{2}}=-\,h{\prime} (1).$$

### Entropy Generation

Entropy generation for the nanobioconvection model is expressed as

43 $$\begin{array}{ccc}{S}_{G} & = & \frac{\alpha }{{T}_{2}^{2}}\left[{\left(\frac{\partial T}{\partial r}\right)}^{2}+{\left(\frac{\partial T}{\partial z}\right)}^{2}\right]+\frac{{\mu }_{nf}}{{T}_{2}}\left[2{\left(\frac{\partial u}{\partial r}\right)}^{2}+2\frac{{u}^{2}}{{r}^{2}}+2{\left(\frac{\partial w}{\partial z}\right)}^{2}\right.\left.{\left(\frac{\partial v}{\partial z}\right)}^{2}+{\left(\frac{\partial u}{\partial z}\right)}^{2}+{\left(r\frac{\partial }{\partial r}\left(\frac{v}{r}\right)\right)}^{2}\right]\\  &  & +\,\frac{\sigma }{{T}_{2}}{B}_{0}^{2}({u}^{2}+{v}^{2})+\frac{RD}{C}\left[{\left(\frac{\partial C}{\partial r}\right)}^{2}+{\left(\frac{\partial C}{\partial z}\right)}^{2}\right]+\frac{RD}{N}\left[{\left(\frac{\partial N}{\partial r}\right)}^{2}+{\left(\frac{\partial N}{\partial z}\right)}^{2}\right]\\  &  & +\,\frac{RD}{{T}_{2}}\left[\frac{\partial C}{\partial r}\frac{\partial T}{\partial r}+\frac{\partial C}{\partial z}\frac{\partial T}{\partial z}\right]+\frac{RD}{{T}_{2}}\left[\frac{\partial N}{\partial r}\frac{\partial T}{\partial r}+\frac{\partial N}{\partial z}\frac{\partial T}{\partial z}\right],\end{array}$$

where *R* is the ideal gas constant and *D* is the diffusivity.

Characteristic entropy generation rate is expressed as

44 $${S}_{0}=\frac{\alpha {(\Delta T)}^{2}{\Omega }_{1}}{{\nu }_{nf}{T}_{2}^{2}}.$$

Applying values from Eq. (17) to Eq. (43), entropy generation number ${N}_{G}=\frac{{S}_{G}}{{S}_{0}}$ becomes

45 $$\begin{array}{lcc}{N}_{G}(\zeta ) & = & \beta {(\theta {\prime} )}^{2}+Br[4{(f{\prime} )}^{2}-4A{(f{\prime} )}^{2}{(g{\prime} )}^{2}+{B}_{1}{(f{\prime} )}^{2}]+M[{(f{\prime} )}^{2}+{(g)}^{2}]+{\gamma }_{1}{(\phi {\prime} )}^{2}+{\gamma }_{2}{(h{\prime} )}^{2}\\  &  & +{\gamma }_{3}\theta {\prime} \phi {\prime} +{\gamma }_{4}\theta {\prime} h{\prime} ,\end{array}$$

where $\beta =\frac{\Delta T}{{T}_{2}}$ is known as the temperature difference parameter, $Br=\frac{{\mu }_{nf}{r}^{2}{({\Omega }_{1})}^{2}}{\alpha \Delta T}$ represents the Brinkman number, $A=\frac{r}{{H}^{2}}$ and ${B}_{1}=\frac{{r}^{2}}{{H}^{2}}$ are some dimensionless parameters, ${\gamma }_{1}=\frac{RD{T}_{2}^{2}({C}_{2}-{C}_{1})}{\alpha {H}^{2}{(\Delta T)}^{2}C}$, ${\gamma }_{2}=\frac{RD{T}_{2}^{2}({N}_{2}-{N}_{1})}{\alpha {H}^{2}{(\Delta T)}^{2}N}$, ${\gamma }_{3}=\frac{RD{T}_{2}({C}_{2}-{C}_{1})}{\alpha {H}^{2}{(\Delta T)}^{2}}$, and ${\gamma }_{4}=\frac{RD{T}_{2}({N}_{2}-{N}_{1})}{\alpha {H}^{2}{(\Delta T)}^{2}}$ are the parameters due to diffusivity of nanoparticles and gyrotactic microorganisms.

The Bejan number is represented as

46 $$Be=\frac{Entropy\ generation\ due\ to\ heat,\ mass\ and\ gyrotactic\ microorganisms\ flow}{Total\ entropy\ generation}.$$

After simplification, Eq. (46) assumes the form

47 $$Be=\frac{\beta {(\theta {\prime} )}^{2}+{\gamma }_{1}{(\phi {\prime} )}^{2}+{\gamma }_{2}{(h{\prime} )}^{2}+{\gamma }_{3}\theta {\prime} \phi {\prime} +{\gamma }_{4}\theta {\prime} h{\prime} }{\beta {(\theta {\prime} )}^{2}+Br[4{(f{\prime} )}^{2}-4A{(f{\prime} )}^{2}{(g{\prime} )}^{2}+B{(f{\prime} )}^{2}]+M[{(f{\prime} )}^{2}+{(g)}^{2}]+{\gamma }_{1}{(\phi {\prime} )}^{2}+{\gamma }_{2}{(h{\prime} )}^{2}+{\gamma }_{3}\theta {\prime} \phi {\prime} +{\gamma }_{4}\theta {\prime} h{\prime} }.$$

## Computation Methodology

Liao^51^ proposed Homotopy Analysis Method (HAM) to solve linear, nonlinear differential equations including algebraic, ordinary differential, partial differential and differential-difference equations. It provides the best solutions and it has been proved that its solution is close to exact solution. HAM has a great variety and has some superior features over other used methods since the other used methods (for example perturbation methods) largely depend on small/large parameters where the convergence of series solution is not found easily or exactly. In HAM, a homotopy technique is used with an embedding parameter which is considered as small so the original nonlinear problem is converted into an infinite number of linear problems without using the perturbation methods.

Some superior qualities of HAM can be enumerated as

(i)        Perturbation methods do not work when both cases of small or large parameter occur while HAM works on homotopic deformation engaging initial guess leading to final outcome.

(ii)      In other methods convergence of solutions is very difficult to achieve while HAM uses a proper mechanisms for the convergence of solution (like in the present problem ℏ is the convergence control parameter and the convergence of solution is achieved very easily in Table 3).

**Table 3:** Convergence of the homotopy solution for different order of approximation.

Order of approximation	− *f* ^*″*^(0)	${\boldsymbol{-}}{\boldsymbol{g}}{\boldsymbol{{\prime} }}$(0)	− ${\boldsymbol{\theta }}{\boldsymbol{{\prime} }}$(0)
1	2.12234561	0.97231567	1.77546831
6	2.12234562	0.97231568	1.77546835
11	2.12234563	0.97231569	1.77546838
16	2.12234563	0.97231569	1.77546838
21	2.12234564	0.97231569	1.77546838
26	2.12234564	0.97231569	1.77546838
31	2.12234564	0.97231569	1.77546838
36	2.12234564	0.97231569	1.77546838
41	2.12234564	0.97231569	1.77546838
42	2.12234564	0.97231569	1.77546838

(iii)    HAM is adjusted i. e. if it is needed to generate solutions in the form of polynomials, exponential or of trigonometric forms, then the base function is adjusted accordingly.

Applying HAM, the initial approximations and auxiliary linear operators are chosen as

48 $${f}_{0}(\zeta )={k}_{1}\zeta -(2{k}_{1}+{k}_{2}){\zeta }^{2}+({k}_{1}+{k}_{2}){\zeta }^{3},\ {g}_{0}(\zeta )=1-\zeta +\Omega \zeta ,\ {\theta }_{0}(\zeta )=1-\zeta ,\ {\phi }_{0}(\zeta )=1-\zeta ,\ {h}_{0}(\zeta )=1-\zeta ,$$

49 $${L}_{f}=f{\prime\prime} {\prime\prime} ,\ \ {{\boldsymbol{L}}}_{g}={g}^{{\prime\prime} },\ \ {{\boldsymbol{L}}}_{\theta }={\theta }^{{\prime\prime} },\ \ {{\boldsymbol{L}}}_{\phi }={\phi }^{{\prime\prime} },\ \ {{\boldsymbol{L}}}_{h}={h}^{{\prime\prime} }$$

characterizing

50 $$\begin{array}{ccc}{{\boldsymbol{L}}}_{f}[{C}_{1}+{C}_{2}\zeta +{C}_{3}{\zeta }^{2}+{C}_{4}{\zeta }^{3}] & = & 0,\ {{\boldsymbol{L}}}_{g}[{C}_{5}+{C}_{6}\zeta ]=0,\ {{\boldsymbol{L}}}_{\theta }[{C}_{7}+{C}_{8}\zeta ]=0,\\  &  & \hspace{4.99878pt}{{\boldsymbol{L}}}_{\phi }[{C}_{9}+{C}_{10}\zeta ]=0,\ {{\boldsymbol{L}}}_{h}[{C}_{11}+{C}_{12}\zeta ]=0,\end{array}$$

evidently *C*_*i*_(*i* = 1–12) are the arbitrary constants.

### Zeroth-order deformation problems

Considering

51 $${\aleph }_{f}[f(\zeta ,q),g(\zeta ,q)]=\frac{{\partial }^{4}f(\zeta ,q)}{\partial {\zeta }^{4}}+Re\left[2f(\zeta ,q)\frac{{\partial }^{3}f(\zeta ,q)}{\partial {\zeta }^{3}}+2g(\zeta ,q)\frac{\partial g(\zeta ,q)}{\partial \zeta }-M\frac{{\partial }^{2}f(\zeta ,q)}{\partial {\zeta }^{2}}\right],$$

52 $${\aleph }_{g}[f(\zeta ,q),g(\zeta ,q)]=\frac{{\partial }^{2}g(\zeta ,q)}{\partial {\zeta }^{2}}+Re\left[2f(\zeta ,q)\frac{\partial g(\zeta ,q)}{\partial \zeta }-M\frac{\partial g(\zeta ,q)}{\partial \zeta }\right],$$

53 $$\begin{array}{ccc}{\aleph }_{\theta }[f(\zeta ,q),g(\zeta ,q),\theta (\zeta ,q)] & = & \frac{{\partial }^{2}\theta (\zeta ,q)}{\partial {\zeta }^{2}}+\,PrRe\left[2f(\zeta ,q)\frac{\partial \theta (\zeta ,q)}{\partial \zeta }+MEc{\left(\frac{\partial f(\zeta ,q)}{\partial \zeta }\right)}^{2}\right.\\  &  & \left.+{(g(\zeta ,q))}^{2}+Nb\frac{\partial \theta (\zeta ,q)}{\partial \zeta }\frac{\partial \phi (\zeta ,q)}{\partial \zeta }+Nt{\left(\frac{\partial \theta (\zeta ,q)}{\partial \zeta }\right)}^{2}\right],\end{array}$$

54 $${\aleph }_{\phi }[f(\zeta ,q),\phi (\zeta ,q)]=\frac{{\partial }^{2}\phi (\zeta ,q)}{\partial {\zeta }^{2}}+Re\left[2Lef(\zeta ,q)\frac{\partial \phi (\zeta ,q)}{\partial \zeta }+\frac{Nt}{Nb}\frac{\partial \theta (\zeta ,q)}{\partial \zeta }\right],$$

55 $${\aleph }_{h}[f(\zeta ,q),\phi (\zeta ,q),h(\zeta ,q)]=\frac{{\partial }^{2}h(\zeta ,q)}{\partial {\zeta }^{2}}+Re\left[2Scf(\zeta ,q)\frac{\partial h(\zeta ,q)}{\partial \zeta }+Pe\left(\frac{\partial h(\zeta ,q)}{\partial \zeta }\frac{\partial \phi (\zeta ,q)}{\partial \zeta }-h(\zeta ,q)\frac{{\partial }^{2}\phi (\zeta ,q)}{\partial {\zeta }^{2}}\right)\right],$$

where ℵ is the nonlinear operator and *q* is an embedding parameter such that *q*  ∈  [0, 1].

Further

56 $$(1-q){{\boldsymbol{L}}}_{f}[f(\zeta ,q)-{f}_{0}(\zeta )]=q{\hslash }_{f}{\aleph }_{f}[f(\zeta ,q),g(\zeta ,q)],$$

57 $$(1-q){{\boldsymbol{L}}}_{g}[g(\zeta ,q)-{g}_{0}(\zeta )]=q{\hslash }_{g}{\aleph }_{g}[f(\zeta ,q),g(\zeta ,q)],$$

58 $$(1-q){{\boldsymbol{L}}}_{\theta }[\theta (\zeta ,q)-{\theta }_{0}(\zeta )]=q{\hslash }_{\theta }{\aleph }_{\theta }[f(\zeta ,q),g(\zeta ,q),\theta (\zeta ,q),\phi (\zeta ,q)],$$

59 $$(1-q){{\boldsymbol{L}}}_{\phi }[\phi (\zeta ,q)-{\phi }_{0}(\zeta )]=q{\hslash }_{\phi }{\aleph }_{\phi }[f(\zeta ,q),\theta (\zeta ,q),\phi (\zeta ,q)],$$

60 $$(1-q){{\boldsymbol{L}}}_{h}[h(\zeta ,q)-{h}_{0}(\zeta )]=q{\hslash }_{h}{\aleph }_{h}[f(\zeta ,q),\phi (\zeta ,q),h(\zeta ,q)],$$

where *ℏ*_*f*_, *ℏ*_*g*_, *ℏ*_*θ*_, *ℏ*_*ϕ*_, and *ℏ*_*h*_ are used for the auxiliary non-zero parameters.

The boundary conditions for Eqs. (56–60) are respectively

61 $$f(0,q)=0,\ f{\prime} (0,q)={k}_{1},\ f(1,q)=0,\ f{\prime} (1,q)={k}_{2},$$

62 $$g(0,q)=1,\ g(1,q)=\Omega ,$$

63 $$\theta (0,q)=1,\ \ \theta (1,q)=0,$$

64 $$\phi (0,q)=1,\ \ \phi (1,q)=0,$$

65 $$h(0,q)=1,\ h(1,q)=0.$$

For *q* = 0 and *q* = 1, the following results are obtained

66 $$q=0\Rightarrow f(\zeta ,0)={f}_{0}(\zeta )\quad and\quad q=1\Rightarrow f(\zeta ,1)=f(\zeta ),$$

67 $$q=0\Rightarrow g(\zeta ,0)={g}_{0}(\zeta )\quad and\quad q=1\Rightarrow g(\zeta ,1)=g(\zeta ),$$

68 $$q=0\Rightarrow \theta (\zeta ,0)={\theta }_{0}(\zeta )\quad and\quad q=1\Rightarrow \theta (\zeta ,1)=\theta (\zeta ),$$

69 $$q=0\Rightarrow \phi (\zeta ,0)={\phi }_{0}(\zeta )\quad and\quad q=1\Rightarrow \phi (\zeta ,1)=\phi (\zeta ),$$

70 $$q=0\Rightarrow h(\zeta ,0)={h}_{0}(\zeta )\quad and\quad q=1\Rightarrow h(\zeta ,1)=h(\zeta ).$$

*f*(*ζ*, *q*) is made *f*_0_(*ζ*) to *f*(*ζ*) when *q* has the values from 0 to 1. *g*(*ζ*, *q*) is made *g*_0_(*ζ*) to *g*(*ζ*) when *q* has the values from 0 to 1. *θ*(*ζ*, *q*) is made *θ*_0_(*ζ*) to *θ*(*ζ*) when *q* has the values from 0 to 1, *ϕ*(*ζ*, *q*) is made *ϕ*_0_(*ζ*) to *ϕ*(*ζ*) for *q* retaining the values from 0 to 1. Similarly *h*(*ζ*, *q*) is made *h*_0_(*ζ*) to *h*(*ζ*) when *q* has the values from 0 to 1.

Introducing Taylor series expansion and Eqs. (66–70), the simplifications are

71 $$f(\zeta ,q)={f}_{0}(\zeta )+\mathop{\sum }\limits_{m=1}^{\infty }\ {f}_{m}(\zeta ){q}^{m},\quad where\quad {f}_{m}(\zeta )=\frac{1}{m!}\frac{{\partial }^{m}f(\zeta ,q)}{\partial {q}^{m}}{| }_{q=0},$$

72 $$g(\zeta ,q)={g}_{0}(\zeta )+\mathop{\sum }\limits_{m=1}^{\infty }{g}_{m}(\zeta ){q}^{m},\quad where\quad {g}_{m}(\zeta )=\frac{1}{m!}\frac{{\partial }^{m}g(\zeta ,q)}{\partial {q}^{m}}{| }_{q=0},$$

73 $$\theta (\zeta ,q)={\theta }_{0}(\zeta )+\mathop{\sum }\limits_{m=1}^{\infty }{\theta }_{m}(\zeta ){q}^{m},\quad where\quad {\theta }_{m}(\zeta )=\frac{1}{m!}\frac{{\partial }^{m}\theta (\zeta ,q)}{\partial {q}^{m}}{| }_{q=0},$$

74 $$\phi (\zeta ,q)={\phi }_{0}(\zeta )+\mathop{\sum }\limits_{m=1}^{\infty }{\phi }_{m}(\zeta ){q}^{m},\quad where\quad {\phi }_{m}(\zeta )=\frac{1}{m!}\frac{{\partial }^{m}\phi (\zeta ,q)}{\partial {q}^{m}}{| }_{q=0},$$

75 $$h(\zeta ,q)={h}_{0}(\zeta )+\mathop{\sum }\limits_{m=1}^{\infty }{h}_{m}(\zeta ){q}^{m},\quad where\quad {h}_{m}(\zeta )=\frac{1}{m!}\frac{{\partial }^{m}h(\zeta ,q)}{\partial {q}^{m}}{| }_{q=0}.$$

The convergence of the series is closely related to *ℏ*_*f*_, *ℏ*_*g*_, *ℏ*_*θ*_, *ℏ*_*ϕ*_ and *ℏ*_*h*_. Let *ℏ*_*f*_, *ℏ*_*g*_, *ℏ*_*θ*_, *ℏ*_*ϕ*_ and *ℏ*_*h*_ are chosen in a manner that the series in Eqs. (71–75) converge at *q* = 1, so Eqs. (71–75) provide

76 $$f(\zeta )={f}_{0}(\zeta )+\mathop{\sum }\limits_{m=1}^{\infty }{f}_{m}(\zeta ),$$

77 $$g(\zeta )={g}_{0}(\zeta )+\mathop{\sum }\limits_{m=1}^{\infty }{g}_{m}(\zeta ),$$

78 $$\theta (\zeta )={\theta }_{0}(\zeta )+\mathop{\sum }\limits_{m=1}^{\infty }{\theta }_{m}(\zeta ),$$

79 $$\phi (\zeta )={\phi }_{0}(\zeta )+\mathop{\sum }\limits_{m=1}^{\infty }{\phi }_{m}(\zeta ),$$

80 $$h(\zeta )={h}_{0}(\zeta )+\mathop{\sum }\limits_{m=1}^{\infty }{h}_{m}(\zeta ).$$

### mth order deformation problems

For Eqs. (56) and (61), the mth order deformation is

81 $${{\boldsymbol{L}}}_{f}[{f}_{m}(\zeta )-{\chi }_{m}\ {f}_{m-1}(\zeta )]={\hslash }_{f}{{\mathfrak{R}}}_{m}^{f}(\zeta ),$$

82 $${f}_{m}(0)=0,\ \ {f}_{m}(1)=0,\ \ {f}_{m}^{{\prime} }(0)=0,\ \ {f}_{m}^{{\prime} }(1)=0,$$

83 $${{\mathfrak{R}}}_{m}^{f}(\zeta )={f}_{m-1}^{{\prime\prime} {\prime\prime} }+Re[{\sum }_{k=o}^{m-1}{f}_{m-1-k}{f}_{k}^{{\prime\prime} {\prime} }+2{g}_{m-1-k}\,{g}_{k}^{{\prime} }-M{f}_{m-1}^{{}^{{\prime\prime} }}].$$

For Eqs. (57) and (62), the mth order deformation is

84 $${{\boldsymbol{L}}}_{g}[{g}_{m}(\zeta )-{\chi }_{m}{g}_{m-1}(\zeta )]={\hslash }_{g}{{\mathfrak{R}}}_{m}^{g}(\zeta ),$$

85 $${g}_{m}(0)=0,\ \ {g}_{m}(1)=0,$$

86 $${{\mathfrak{R}}}_{m}^{g}(\zeta )={g}_{m-1}^{{}^{{\prime\prime} }}+Re[{\sum }_{k=o}^{m-1}2{f}_{m-1-k}{g}_{k}^{{\prime} }-M{g}_{m-1}^{{\prime} }].$$

For Eqs. (58) and (63), the mth order deformation is

87 $${{\boldsymbol{L}}}_{\theta }[{\theta }_{m}(\zeta )-{\chi }_{m}{\theta }_{m-1}(\zeta )]={\hslash }_{\theta }{{\mathfrak{R}}}_{m}^{\theta }(\zeta ),$$

88 $${\theta }_{m}(0)=0,\ \ {\theta }_{m}(1)=0,$$

89 $$\begin{array}{cll}{{\mathfrak{R}}}_{m}^{\theta }(\zeta ) & = & {\theta }_{m-1}^{{}^{{\prime\prime} }}+PrRe[2{\sum }_{k=o}^{m-1}{f}_{m-1-k}{\theta }_{k}^{{\prime} }]+PrReMEc[{\sum }_{k=o}^{m-1}{f}_{m-1-k}^{{\prime} }{f}_{k}^{{\prime} }+{\sum }_{k=o}^{m-1}{g}_{m-1-k}{g}_{k}]\ \\  &  & \hspace{4.99878pt}+PrRe[Nb{\sum }_{k=o}^{m-1}{\theta }_{m-1-k}^{{\prime} }{\phi }_{k}^{{\prime} }+Nt{\sum }_{k=o}^{m-1}{\theta }_{m-1-k}^{{\prime} }{\theta }_{k}^{{\prime} }].\end{array}$$

For Eqs. (59) and (64), the mth order deformation is

90 $${{\boldsymbol{L}}}_{\phi }[{\phi }_{m}(\zeta )-{\chi }_{m}{\phi }_{m-1}(\zeta )]={\hslash }_{\phi }{{\mathfrak{R}}}_{m}^{\phi }(\zeta ),$$

91 $${\phi }_{m}(0)=0,\ \ {\phi }_{m}(1)=0,$$

92 $${{\mathfrak{R}}}_{m}^{\phi }(\zeta )={\phi }_{m-1}^{{}^{{\prime\prime} }}+Re\left[{\sum }_{k=o}^{m-1}2Le{f}_{m-1-k}{\phi }_{k}^{{\prime} }+\frac{Nt}{Nb}{\theta }_{m-1}^{{\prime} }\right].$$

For Eqs. (60) and (65), the mth order deformation is

93 $${{\boldsymbol{L}}}_{h}[{h}_{m}(\zeta )-{\chi }_{m}{h}_{m-1}(\zeta )]={\hslash }_{h}{{\mathfrak{R}}}_{m}^{h}(\zeta ),$$

94 $${h}_{m}(0)=0,\ \ {h}_{m}(1)=0,$$

95 $${{\mathfrak{R}}}_{m}^{h}(\zeta )={h}_{m-1}^{{}^{{\prime\prime} }}+Re[{\sum }_{k=o}^{m-1}2Sc{f}_{m-1-k}{h}_{k}^{{\prime} }+Pe({\sum }_{k=o}^{m-1}{h}_{m-1-k}^{{\prime} }{\phi }_{k}^{{\prime} }-{\sum }_{k=o}^{m-1}{h}_{m-1-k}{\phi }_{k}^{{}^{{\prime\prime} }})].$$

96 $${\chi }_{m}=\left\{\begin{array}{ll}0, & m\le 1\\ 1, & m > 1.\end{array}\right.$$

Combining the special solutions ${f}_{m}^{\ast }(\zeta )$, ${g}_{m}^{\ast }(\zeta )$, ${\theta }_{m}^{\ast }(\zeta )$, ${\phi }_{m}^{\ast }(\zeta )$ and ${h}_{m}^{\ast }(\zeta )$, the general solutions of Eqs. (81), (84), (87), (90) and (93) are

97 $${f}_{m}(\zeta )={f}_{m}^{\ast }(\zeta )+{C}_{1}+{C}_{2}\zeta +{C}_{3}{\zeta }^{2}+{C}_{4}{\zeta }^{3},$$

98 $${g}_{m}(\zeta )={g}_{m}^{\ast }(\zeta )+{C}_{5}+{C}_{6}\zeta ,$$

99 $${\theta }_{m}(\zeta )={\theta }_{m}^{\ast }(\zeta )+{C}_{7}+{C}_{8}\zeta ,$$

100 $${\phi }_{m}(\zeta )={\phi }_{m}^{\ast }(\zeta )+{C}_{9}+{C}_{10}\zeta ,$$

101 $${h}_{m}(\zeta )={h}_{m}^{\ast }(\zeta )+{C}_{11}+{C}_{12}\zeta .$$

## Results and discussion

Results are obtained for the non-linear differential Eqs. in (19,21–26) through the application of MATHEMATICA. Equations (32, 33, 36, 39, 42) and (45) are solved with the obtained HAM solution for discussing skin friction coefficients, Nusselt numbers, Sherwood numbers, motile microorganisms fluxes and entropy generation. The geometry of the problem is demonstrated in Fig. 1. Following Liao^51^, the valid ℏ-curves for *f*(*ζ*), *g*(*ζ*), *θ*(*ζ*), *ϕ*(*ζ*) and *h*(*ζ*) are constructed with the ranges −1.5 ≤ *ℏ*_*f*_ ≤ − 0.4, − 20.0 ≤ *ℏ*_*g*_ ≤ 18.0, − 2.0 ≤ *ℏ*_*θ*_ ≤ 0.0, − 2.0 ≤ *ℏ*_*ϕ*_ ≤ 0.0 and − 2.5 ≤ *ℏ*_*h*_ ≤ 0.5 in Figs. 2–6. The effects of relevant parameters on the respective profiles are displayed in Figs. 7–39. Convergence of the HAM solution is shown through Table 3. Comparison of the existing work with published work is presented through Tables 4 and 5.

**Figure 2:**
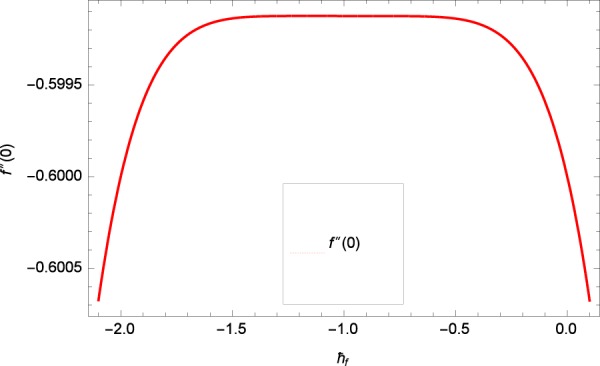
*ℏ*_*f*_ curve of *f*(*ζ*).

**Figure 3:**
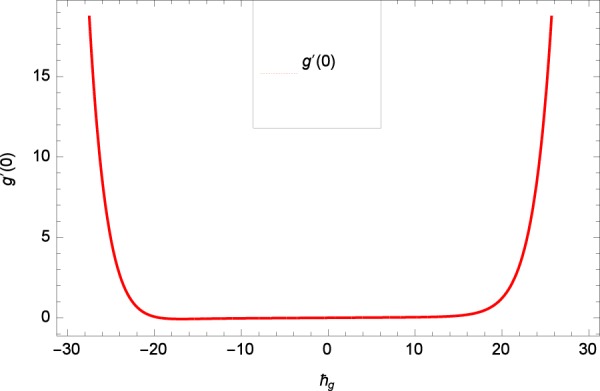
*ℏ*_*g*_ curve of *g*(*ζ*).

**Figure 4:**
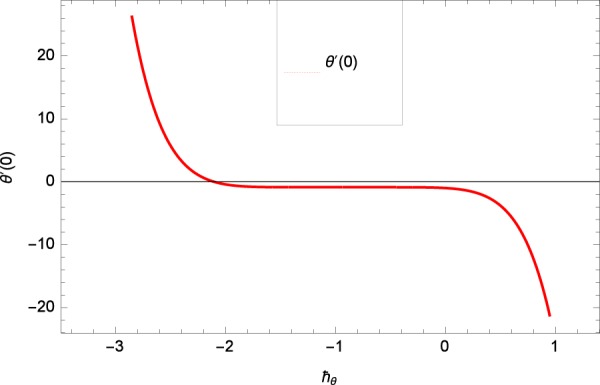
*ℏ*_*θ*_ curve of *θ*(*ζ*).

**Figure 5:**
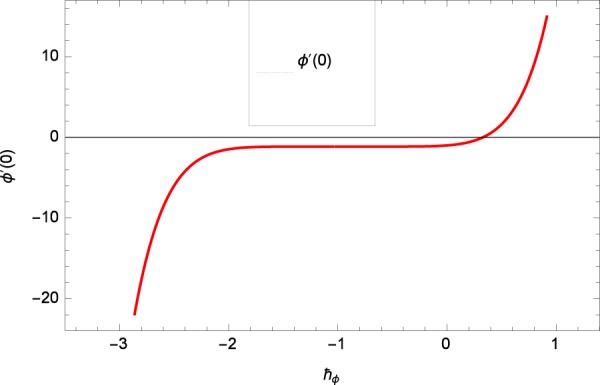
*ℏ*_*ϕ*_ curve of *ϕ*(*ζ*).

**Figure 6:**
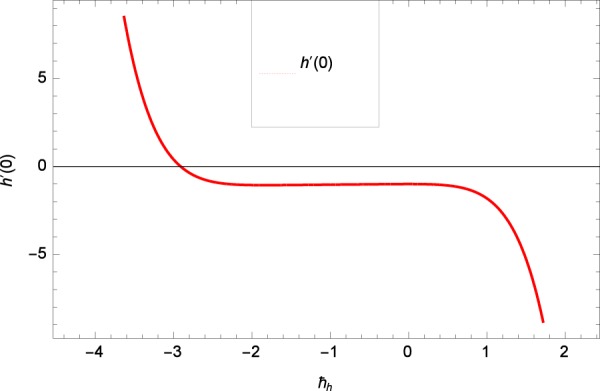
*ℏ*_*h*_ curve of *h*(*ζ*).

**Figure 7:**
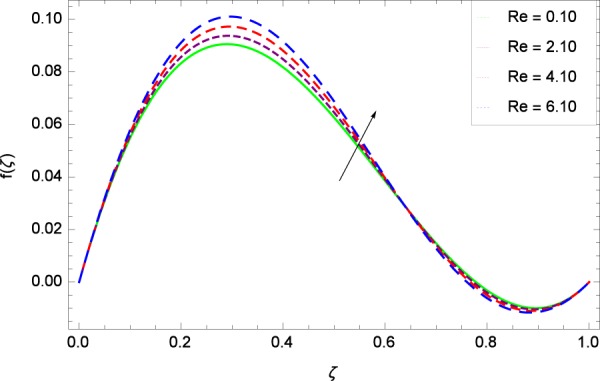
Axial velocity in the consideration of *Re*.

**Figure 8:**
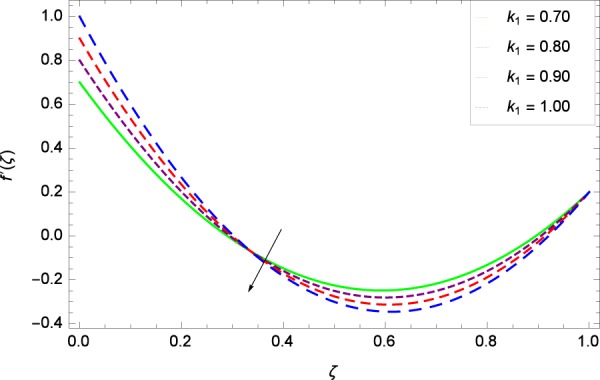
Radial velocity in the consideration of *k*_1_.

**Figure 9:**
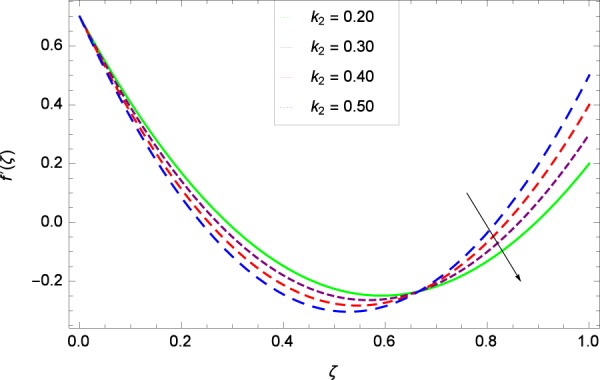
Radial velocity in the consideration of *k*_2_.

**Figure 10:**
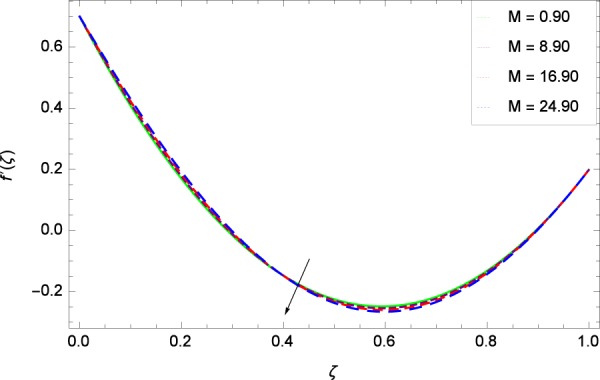
Radial velocity in the consideration of *M*.

**Figure 11:**
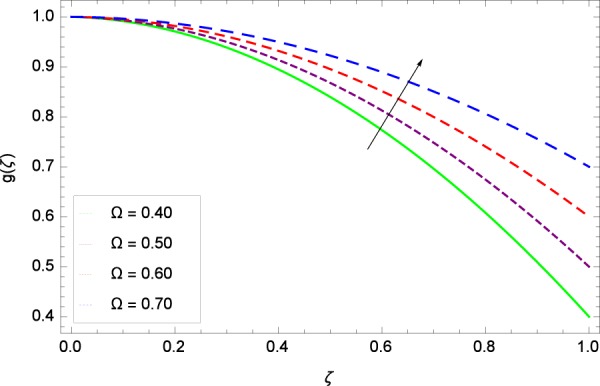
Tangential velocity in the consideration of Ω.

**Figure 12:**
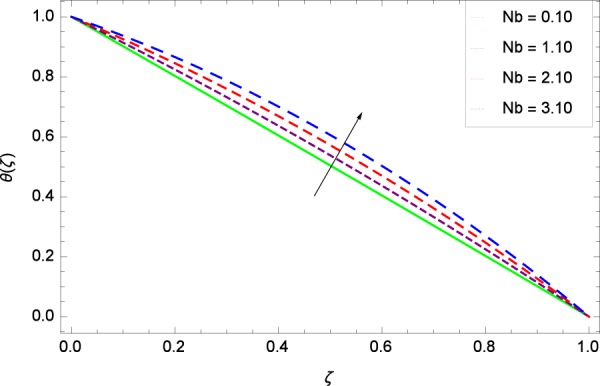
Temperature in the consideration of *Nb*.

**Figure 13:**
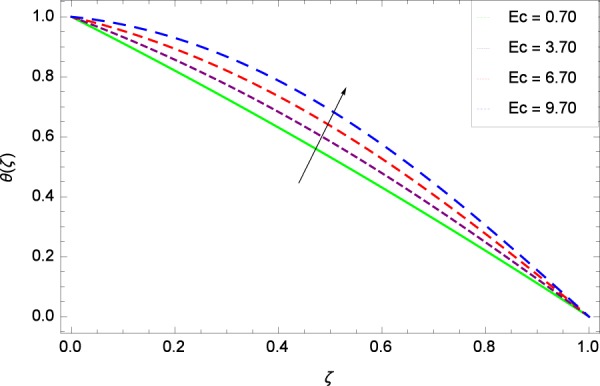
Temperature in the consideration of *Ec*.

**Figure 14:**
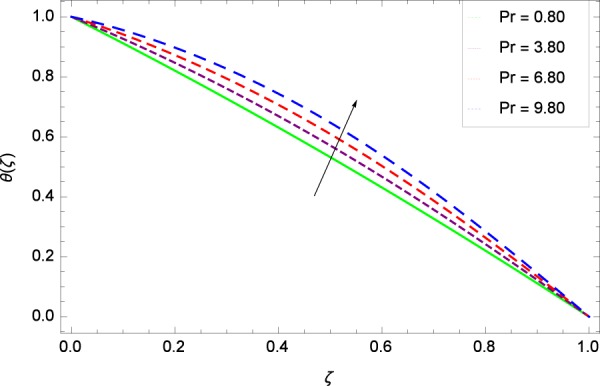
Temperature in the consideration of *Pr*.

**Figure 15:**
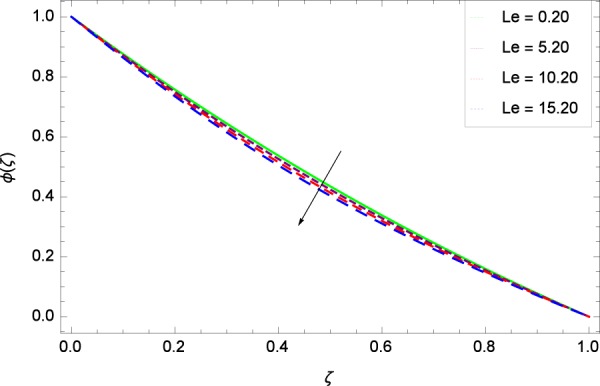
Nanoparticles concentration in the consideration of *Le*.

**Figure 16:**
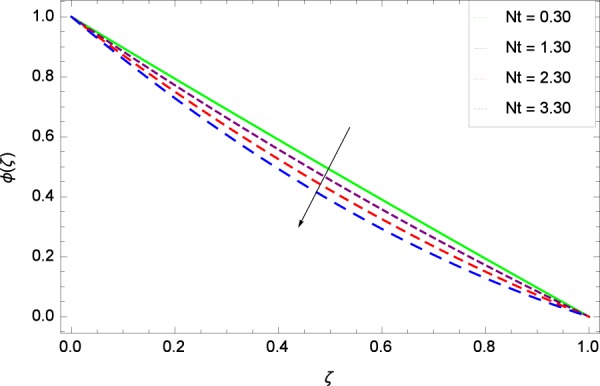
Nanoparticles concentration in the consideration of *Nt*.

**Figure 17:**
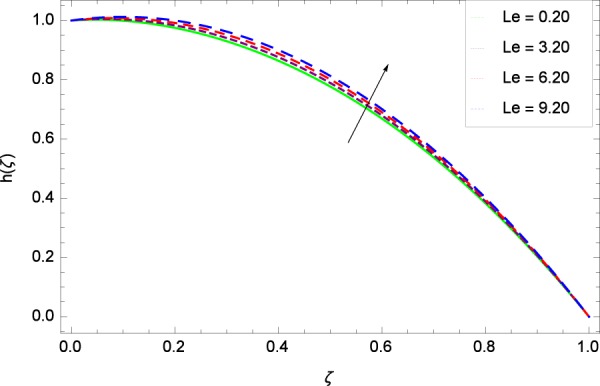
Motile microorganisms concentration in the consideration of *Le*.

**Figure 18:**
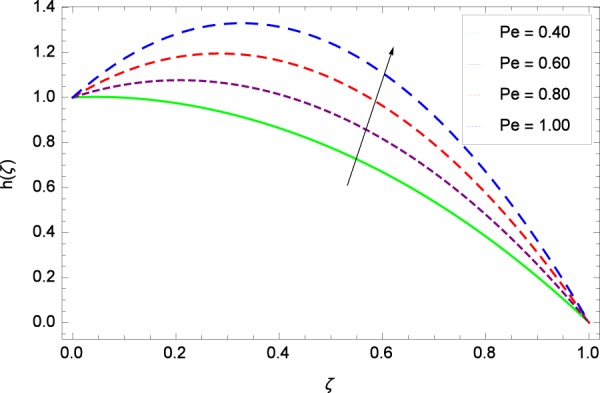
Motile microorganisms concentration in the consideration of *Pe*.

**Figure 19:**
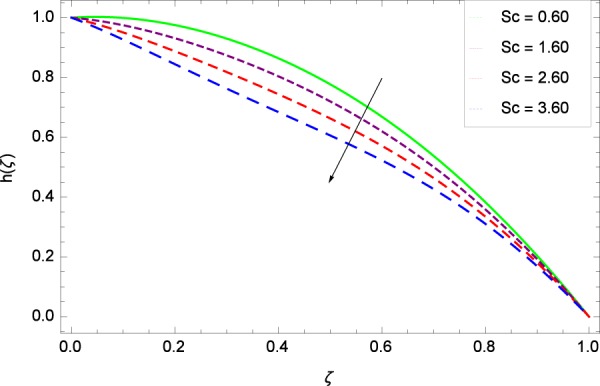
Motile microorganisms concentration in the consideration of *Sc*.

**Figure 20:**
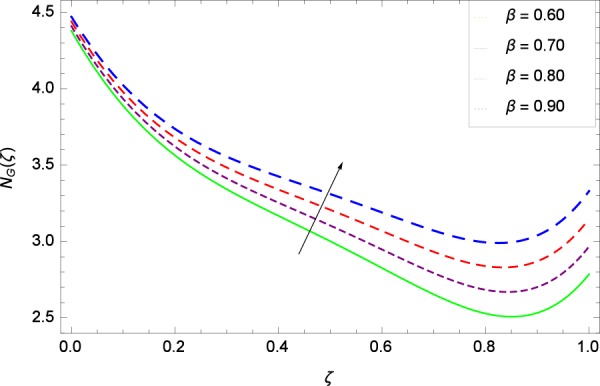
Entropy generation rate in the consideration of *β*.

**Figure 21:**
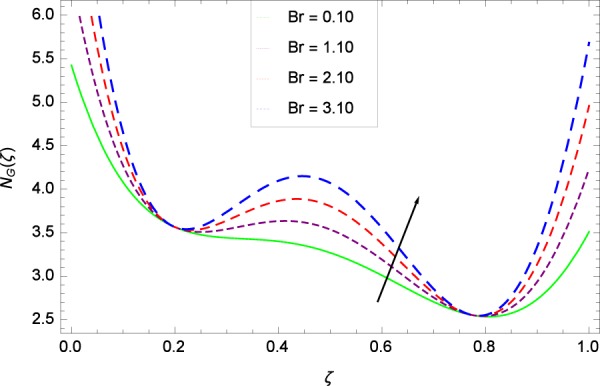
Entropy generation rate in the consideration of *Br*.

**Figure 22:**
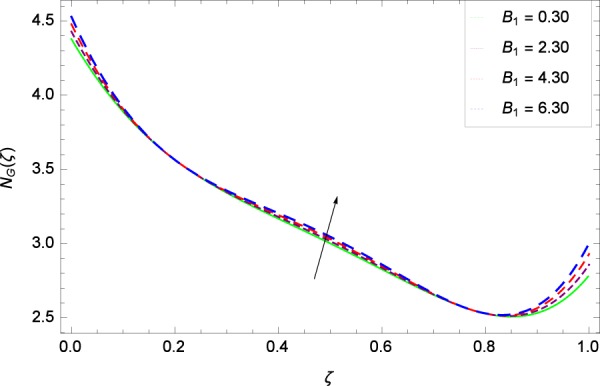
Entropy generation rate in the consideration of *B*_1_.

**Figure 23:**
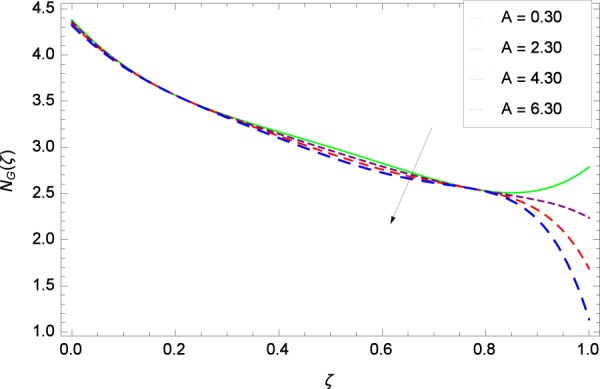
Entropy generation rate in the consideration of *A*.

**Figure 24:**
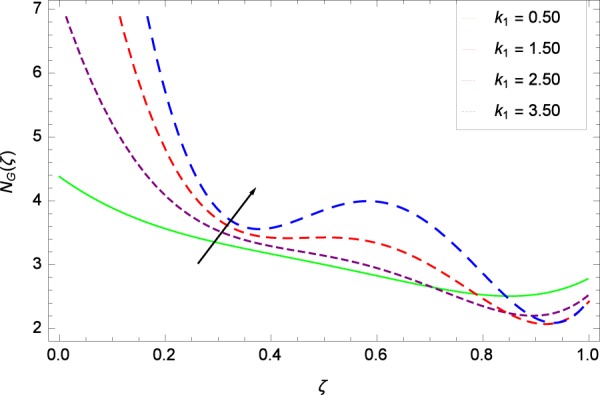
Entropy generation rate in the consideration of *k*_1_.

**Figure 25:**
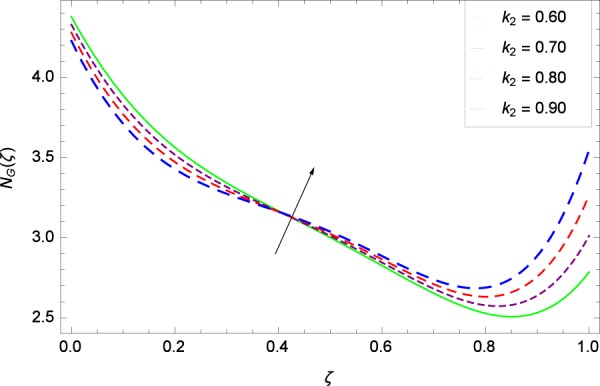
Entropy generation rate in the consideration of *k*_2_.

**Figure 26:**
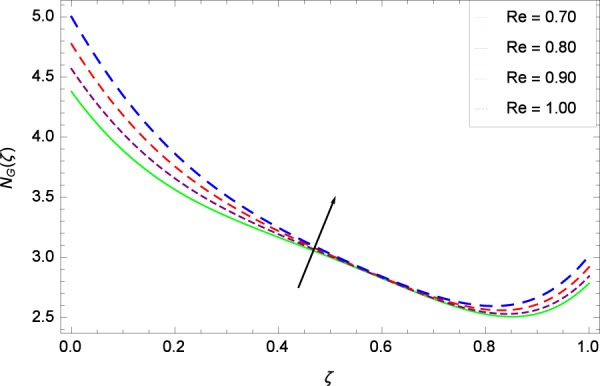
Entropy generation rate in the consideration of *Re*.

**Figure 27:**
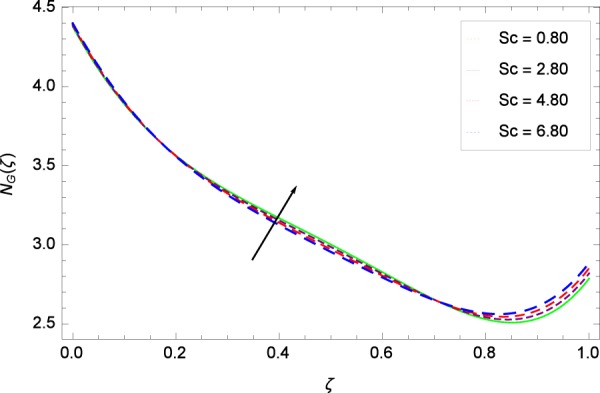
Entropy generation rate in the consideration of *Sc*.

**Figure 28:**
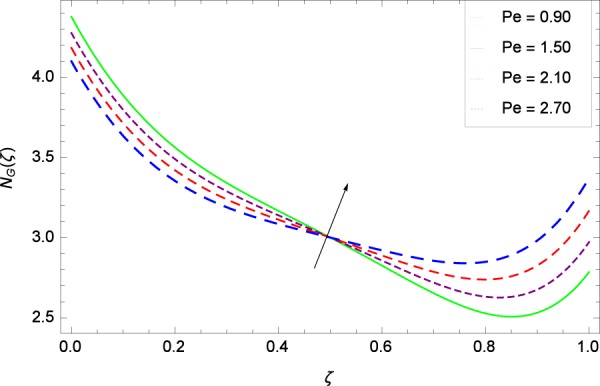
Entropy generation rate in the consideration of *Pe*.

**Figure 29:**
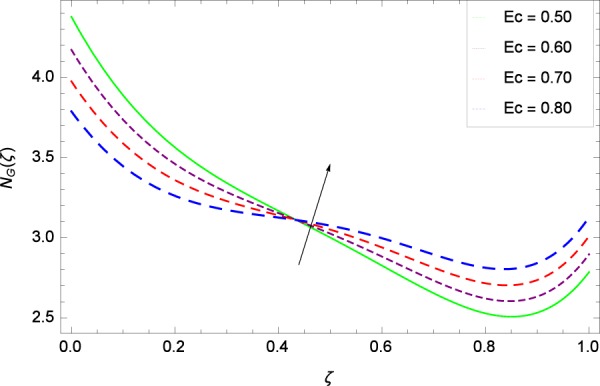
Entropy generation rate in the consideration of *Ec*.

**Figure 30:**
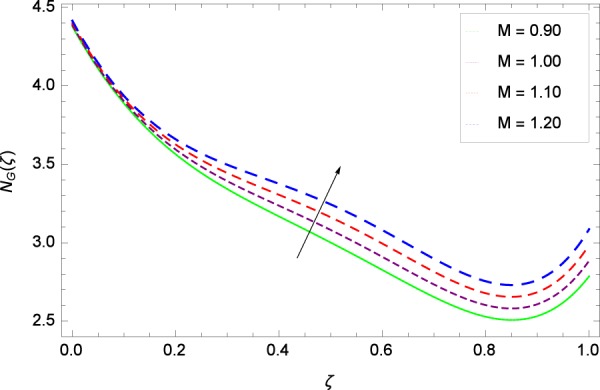
Entropy generation rate in the consideration of *M*.

**Figure 31:**
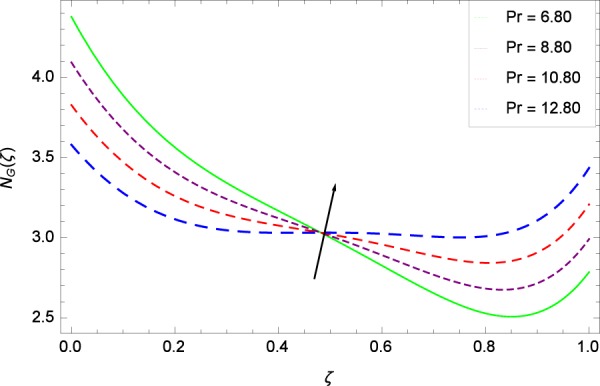
Entropy generation rate in the consideration of *Pr*.

**Figure 32:**
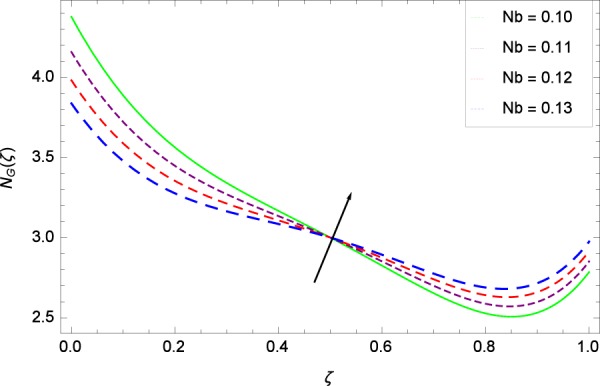
Entropy generation rate in the consideration of *Nb*.

**Figure 33:**
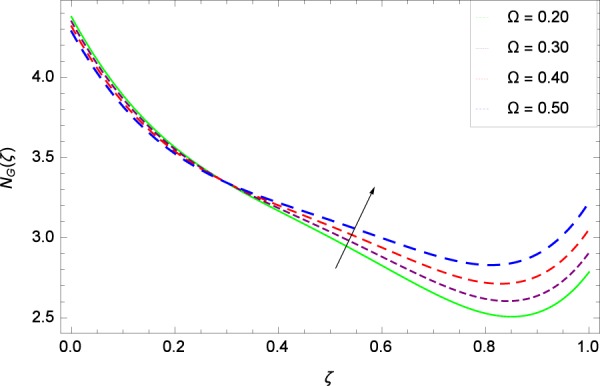
Entropy generation rate in the consideration of Ω.

**Figure 34:**
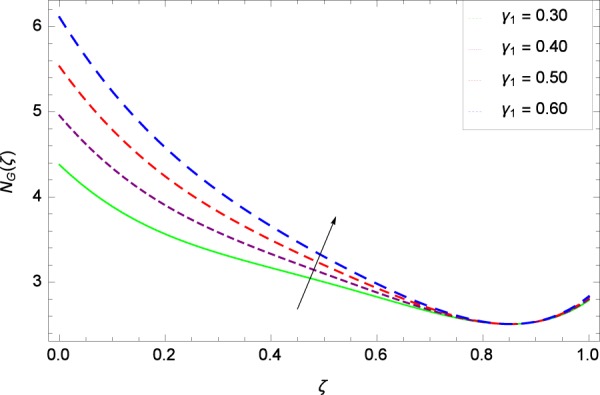
Entropy generation rate in the consideration of *γ*_1_.

**Figure 35:**
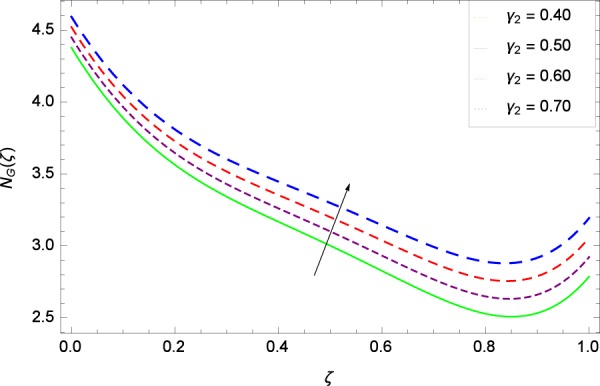
Entropy generation rate in the consideration of *γ*_2_.

**Figure 36:**
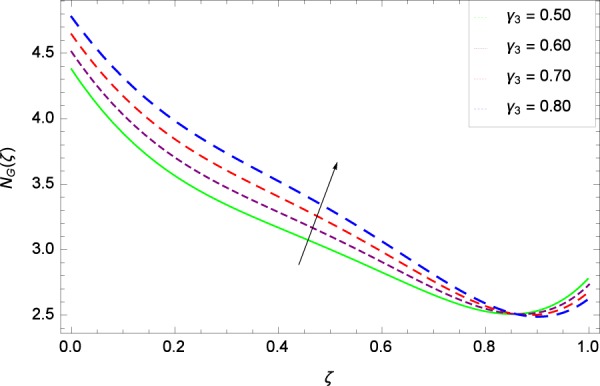
Entropy generation rate in the consideration of *γ*_3_.

**Figure 37:**
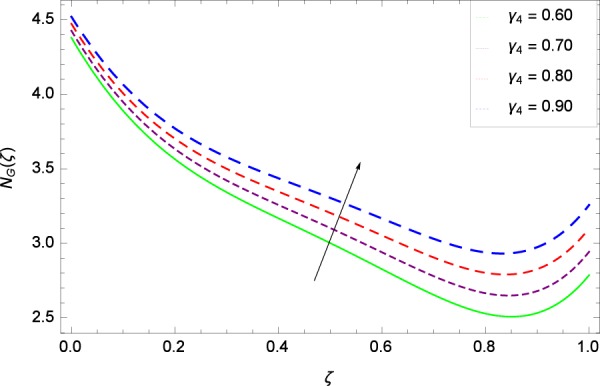
Entropy generation rate in the consideration of *γ*_4_.

**Figure 38:**
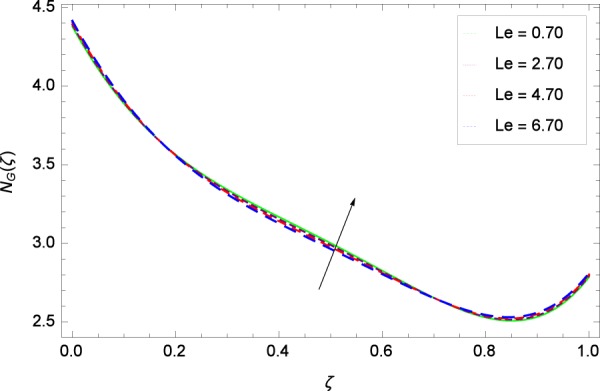
Entropy generation rate in the consideration of *Le*.

**Figure 39:**
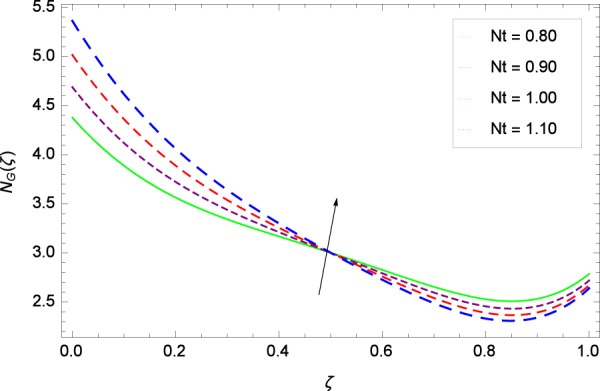
Entropy generation rate in the consideration of *Nt*.

**Table 4:** Comparison of *f* ^*″*^(0) and $g{\prime}$(0) with previous work.

Ω	*f* ^*″*^(0)^38^	*f* ^*″*^(0)(present)	${\boldsymbol{g}}{\boldsymbol{{\prime} }}$(0)^38^	${\boldsymbol{g}}{\boldsymbol{{\prime} }}$(0)(present)
−1.0	0.066663140	0.066663112	2.0009522	2.0009513
−0.8	0.083942070	0.083942039	1.8025885	1.8025824
−0.3	0.10395088	0.10395046	1.3044236	1.3044233
0.0	0.099972208	0.099972211	1.0042776	1.0042775
0.50	0.066634195	0.066634178	0.50261351	0.50261355

**Table 5:** Comparison of $\theta {\prime}$(1), $\phi {\prime}$(1) and $h{\prime}$(1) with previous work.

*Pr*	${\boldsymbol{\theta }}{\boldsymbol{{\prime} }}$(1)^34^	${\boldsymbol{\theta }}{\boldsymbol{{\prime} }}$(1) (present)	${\boldsymbol{\phi }}{\boldsymbol{{\prime} }}$(1)^34^	${\boldsymbol{\phi }}{\boldsymbol{{\prime} }}$(1) (present)	${\boldsymbol{h}}{\boldsymbol{{\prime} }}$(0)^34^	${\boldsymbol{h}}{\boldsymbol{{\prime} }}$(0)(present)
0.01	0.251679	0.251671	−0.251679	−0.251671	2.0009522	2.0009513
1.00	0.247780	0.247789	−0.247780	−0.247789	0.268805	0.268801
3.00	0.239800	0.239801	−0.239800	−0.239801	0.282821	0.282823
5.00	0.231716	0.231715	−0.231716	−0.231715	0.296777	0.296775
7.00	0.223570	0.223571	−0.223570	−0.223571	0.310617	0.310616

### Dynamic role of profiles

Figure 7  shows the effect of Reynolds number *Re* on axial velocity *f*(*ζ*) observing that the enhancement in velocity profile is very much significant with enhancement in Reynolds number. The reflection point in that figure lies in the neighboring of 0.25 where Reynolds number effect turns to decreasing behavior. Graph behavior is similar to the Fig. 3 of the study by Hayat *et al*.^35^ and Fig. 5(a) of the study by Ahmed *et al*.^36^. Figure 8 reveals that radial velocity $f{\prime} (\zeta )$ increases with stretching parameter *k*_1_ in the effectiveness of rotating system. Velocity becomes negative at lower disk due to high stretching. The tendency of the graph is already obtained in Fig. 6 by Hayat *et al*.^35^. Due to centrifugal force, the fluid particles are pushed away in the radial direction. *k*_1_ = 0 shows that the lower disk is unaffected by stretching phenomena. Radial velocity changes its sign near *ζ* = 0.3 which is the inflection point hence the fluid drawn radially inwards due to slow rotating disk and is thrown radially outwards due to fast rotating disk.

The notion in Fig. 9 has the similar result about the parameter *k*_2_ and radial velocity $f{\prime} (\zeta )$. It provides opportunities for bringing back past memory (effect in Fig. 8), getting to know about the profiles. The graph behavior is closely matched with the Fig. 8 in^35^, Fig. 3(c) in^36^ and Fig. 9 in the paper by Khan *et al*.^38^. *k*_2_ = 0 shows that the stretching rate at upper disk is absent. In such situation, the axial velocity component is positive for positive and negative directions of rotation. Therefore, near the lower disk, the radial velocity is positive and negative near the upper disk. It is noted that due to the strong stretching rate (*k*_2_ = 0.20, 0.30, 0.40, 0.50) at upper disk, slow motion is made and the inflection point is shifted towards the fast rotating disk in the radial velocity. Figure 10 shows the effect of magnetic field parameter *M* on radial velocity $f{\prime} (\zeta )$ to decrease the flow. The value of *M* = $\frac{{\sigma }_{f}{B}_{0}^{2}}{{\rho }_{f}{\Omega }_{1}}$ which adheres to the simplification of the nonlinear term ${\sigma }_{f}{B}_{0}^{2}({u}^{2}+{v}^{2})$ in Eq. (12) and also committed to exist as a last negative term −${\sigma }_{f}{B}_{0}^{2}u$ and −${\sigma }_{f}{B}_{0}^{2}v$ each in Eqs. (9) and (10) respectively. So in non-dimensional form (*M*) of these terms does a lot to motion and heating. A drag-like Lorentz force is generated by the application of magnetic field on the electrically conducting fluid. This force has the tendency to slow down the flow around the disk. Figure 10 follows the trend of Figs. 4(c)^36^ and 3^45^. Figure 11 is the result of an effective collaboration between the rotation parameter Ω and the tangential velocity *g*(*ζ*). Rotation paves a long way in improving the motion. Ω is defined in Table 2 as $\Omega =\frac{{\Omega }_{2}}{{\Omega }_{1}}$ which has the basic effective results for rotatory motion. When Ω <  0 i. e. for the negative values of Ω, the direction of motion of both the disks become opposite. Ω = 0 implies that the upper disk has no part in motion and is at rest. Ω >  0 explains that the direction of motion of both the disks is same and in particular, Ω = 1 interprets that both the disks have the same speed and directions. Figure 11 has close resemblance with Fig. 11 of^35^.

It is easy to understand that the directions of rotation of both the disks are important in the motion of both the disks. When both the discs rotate in the same sense then the fluid in the disks rotates with an angular velocity. In particular case, the motion of upper disk is higher compared to the lower disk, the radial flow is inwards near the lower disk and outwards near the upper one. On the other side, if the lower disk rotates faster than upper one, then fluid flows inwards near to the lower disk and outwards near to the upper disk. In both cases, the two disks are attracted to one another which indicates that the pressure between the two disks decreases.

The rotation in the opposite sense of both the disks, a plane exist between the two disks where the tangential velocity has zero magnitude. In such a case, the radial velocity of the fluid is inwards near the plane and outwards in the vicinity of both the disks. At this time, both the disks repel one another which causes to increase the pressure.

The Brownian motion parameter *Nb* projects its influence on temperature *θ*(*ζ*) in Fig. 12. Brownian motion is the core objective of the present system. Nanoparticles and nanofluids community confirm the Brownian motion contribution on real time basis. Brownian motion is the result of random motion of the nanoparticles which causes to increase the temperature. This was also shown by Ahmed *et al*.^36^ in Fig. 8(b). The greater values of *Ec* are used to access the enhanced temperature *θ*(*ζ*) in Fig. 13. The parameter *Ec* is assigned the values 0.70, 3.70, 6.70 and 9.70 which is marked due to the fact that energy is stored in the fluid region as a consequence of dissipation because of viscosity. The system gets the parameter *Pr* by assigning the designated values 0.80, 3.80, 6.80 and 9.80 to enhance the temperature shown through Fig. 14. Physically, thermal diffusivity is reduced with higher values of Prandtl number.

The nanofluid active parameters are Lewis number *Le* and thermophoresis parameter *Nt*. Both *Le* and *Nt* have significant role in mass transfer characteristics. Figure 15 shows that *ϕ*(*ζ*) is reduced with increasing values of Lewis number *Le* to ensure the power of nanoparticles diffusion. Lewis number *Le* is inversely related to the diffusion of nanoparticles. Figure 16 witnesses that the thermophoresis parameter *Nt* decreases the nanoparticle concentration *ϕ*(*ζ*). Enhancement of *Nt* aims not to improve the concentration. Thermophoresis works to push the nanoparticles from high energy state to low energy state on account of using temperature so in the present case temperature is used which affects the concentration. The result of Fig. 16 is authenticated by the consequence of Fig. 6(B) of the work of Rout *et al*.^32^ and Fig. 9 of Ahmed *et al*.^36^.

Heating makes the system more unstable and accelerates the development of bioconvection. Figure 17 depicts that for the increasing values of Lewis number *Le*, the motile microorganisms concentration *h*(*ζ*) enhancement is observed. It is due to the fact that the density and boundary layer thickness of motile microorganisms is increased. Motile microorganisms concentration *h*(*ζ*) is easily enhanced for the prescribed values of *Pe* in Fig. 18. The addiction of such behavior of *Pe* to the surrounding (*h*(*ζ*)) can be comprehended from Eq. (23) $h{\prime\prime} +Re[2Scfh{\prime} +Pe(h{\prime} \phi {\prime} -h\phi {\prime\prime} )]=0$, where strong coupling relation of *Pe* is observed with nanoparticles field *ϕ* and microorganisms concentration field *h*. It is witnessed that as *Pe* is attempting to resume positive values, event causes *h*(*ζ*) to high position. Figure 19 records the evidence of motile microorganisms concentration *h*(*ζ*) and Schmidt number *Sc*. Increasing values of *Sc* reduce the concentration profile *h*(*ζ*).

### Entropy generation rate

Figure 20  illustrates the entropy generation rate *N*_*G*_(*ζ*) and the temperature difference parameter *β* behaviors. It proves that entropy generation rate *N*_*G*_(*ζ*) is enhanced with increasing values of *β*, as there is no challenge to the entropy existence, already reported by Khan *et al*.^38^ in Fig. 25. The view of Fig. 21 is conveying a prompt response to permit that entropy generation is made high due to the positive values of Brinkman number *Br* like in^38^ through Fig. 23. In Fig. 22, the non-dimensional parameter *B*_1_ allows the entropy generation rate *N*_*G*_(*ζ*) to grow large by adding *B*_1_. The other non-dimensional parameter *A* has good and interesting effect, shown in Fig. 23, not to increase *A*-based entropy generation rate *N*_*G*_(*ζ*). So the irreversibility of the thermal system is reduced by adjusting the term *A*. Figure 24 takes several values of stretching parameter *k*_1_ and delivers high entropy generation rate *N*_*G*_(*ζ*) wheres in Fig. 25 for the stretching parameter *k*_2_, the entropy generation rate *N*_*G*_(*ζ*) initially decreases and then increases which has been discussed in a similar way by Fig. 29^38^. Figure 26 projects that Reynolds number *Re* provides incremental values to entropy generation rate *N*_*G*_(*ζ*). Similarly in the other figure, namely Fig. 27, the entropy generation rate *N*_*G*_(*ζ*) is notified for the information that for any quantity of Schmidt number *Sc*, *N*_*G*_(*ζ*) is positively affected. In Fig. 28, it is stated that on the appearance of Peclet number *Pe*, the entropy is growing large. On Fig. 29, the constituents represent the irreversibility rate *N*_*G*_(*ζ*) along with the Eckert number *Ec* enhancement under this figure. Figure 30 shows that there are certain provisions in the magnetic field parameter *M*, mostly four values of *M* which make high the entropy generation rate. Figure 31 reveals that the present forms of entropy generation rate *N*_*G*_(*ζ*) and Prandtl number *Pr* are enhanced.

Figure 32 provides the information that according to said figure, the size of entropy generation rate *N*_*G*_(*ζ*) is maximized for high values of Brownian motion parameter *Nb*. Figure 33 is related to rotation parameter Ω and entropy generation rate *N*_*G*_(*ζ*) possessing maximization in both Ω and *N*_*G*_(*ζ*). The concentration diffusivity parameters *γ*_1_ in Fig. 34 and *γ*_3_ in Fig. 35 respectively are showing the same behaviors. Similarly the decisions are exist for microorganisms concentration diffusivity parameters *γ*_2_ in Fig. 36 and *γ*_4_ in Fig. 37 respectively to enhance the entropy generation rate *N*_*G*_(*ζ*). One of the fundamental parameter *Le* in Fig. 38 increases the entropy generation rate *N*_*G*_(*ζ*). Figure 39 projects in a manner where the thermophoresis parameter *Nt* influence is involved to increase the entropy generation rate *N*_*G*_(*ζ*).

## Conclusions

Buongiorno’s nanofluid model is used to model the problem between two stretchable rotating disks with flow, heat and mass transfer as well as gyrotactic microorganisms and entropy generation. Homotopy analysis method (HAM) is applied to solve the problem and the solution is shown through graphs for the interesting effects of all the embedded parameters.

The findings are summarized as follow.

(1)    Reynolds number *Re* increases the axial velocity *f*(*ζ*). Stretching parameters *k*_1_, *k*_2_ and magnetic field parameter *M* decrease the radial velocity while rotation parameter Ω increases the tangential velocity.

(2)    Brownian motion parameter *Nb*, Eckert number *Ec* and Prandtl number *Pr* increase the temperature.

(3)    Lewis number *Le* and Peclet number *Pe* increase the microorganisms concentration while the Schmidt number *Sc* decreases the same profile.

(4)    Temperature difference parameter *β*, Brinkman number *Br*, non-dimensional constant *B*_1_, stretching parameters *k*_1_ and *k*_2_, Reynolds number *Re*, Schmidt number *Sc*, Peclet number *Pe*, Eckert number *Ec*, magnetic field parameter *M*, Prandtl number *Pr*, Brownian motion parameter *Nb*, rotation parameter Ω, nanoparticles and gyrotactic microorganisms diffusivity parameters *γ*_1_, *γ*_2_, *γ*_3_ and *γ*_4_, Lewis number *Le* and thermophoresis parameter *Nt* increase the entropy generation rate while the non-dimensional constant *A* decreases the same profile.

(5)   Convergence of the HAM solution is shown through Table 3 and close agreement is found in Tables 4 and 5 with the published work.

## Data Availability

All the relevant material is available.
